# Estimativa de incidência de câncer no Brasil e regiões em 2018: aspectos metodológicos

**DOI:** 10.1590/0102-311XPT131623

**Published:** 2024-07-29

**Authors:** Beatriz Cordeiro Jardim, Washington Leite Junger, Regina Paiva Daumas, Gulnar Azevedo e Silva

**Affiliations:** 1 Instituto Nacional de Câncer, Rio de Janeiro, Brasil.; 2 Instituto de Medicinal Social Hesio Cordeiro, Universidade do Estado do Rio de Janeiro, Rio de Janeiro, Brasil.; 3 Escola Nacional de Saúde Pública Sergio Arouca, Fundação Oswaldo Cruz, Rio de Janeiro, Brasil.

**Keywords:** Técnicas de Estimativa, Câncer, Sistemas de Informação em Saúde, Estimation Techniques, Cancer, Health Information Systems, Técnicas de Estimación, Cáncer, Sistemas de Información en Salud

## Abstract

O objetivo deste estudo foi desenvolver metodologia para estimar a incidência de câncer no Brasil e regiões. A partir de dados dos registros de câncer de base populacional (RCBP) e do Sistema de Informações sobre Mortalidade (SIM) foram calculadas razões de incidência e mortalidade (I/M) anuais, tipo de câncer, faixa etária e sexo em cada RCBP. Para estimar as razões I/M por região em 2018, foram aplicados modelos multiníveis longitudinais de Poisson. A estimativa de casos novos de câncer, em 2018, foi calculada aplicando-se as razões I/M estimadas ao número de óbitos corrigidos do SIM ocorridos naquele ano. Norte e Nordeste concentraram as menores razões I/M. Os cânceres de pâncreas, pulmão, fígado e esôfago tiveram as menores razões I/M, enquanto as maiores razões I/M foram estimadas para câncer de tireoide, testículo, próstata e mama feminina. Para 2018, foram estimados 506.462 casos novos de câncer no Brasil. Mama feminina e próstata foram os dois principais tipos de câncer em todas as regiões. No Norte e no Nordeste, destacaram-se os cânceres do colo do útero e de estômago. Diferenças nas razões I/M entre as regiões foram observadas e podem estar relacionadas ao desenvolvimento socioeconômico e ao acesso a serviços de saúde.

## Abstract (en)

The aim of this study was to develop a methodology for estimating cancer incidence in Brazil and its regions. Using data from population-based cancer registries (RCBP, acronym in Portuguese) and the Brazilian Mortality Information System (SIM, acronym in Portuguese), annual incidence/mortality (I/M) ratios were calculated by type of cancer, age group and sex in each RCBP. Poisson longitudinal multilevel models were applied to estimate the I/M ratios by region in 2018. The estimate of new cancer cases in 2018 was calculated by applying the estimated I/M ratios to the number of SIM-corrected deaths that occurred that year. North and Northeast concentrated the lowest I/M ratios. Pancreatic, lung, liver and esophageal cancers had the lowest I/M ratios, whereas the highest were estimated for thyroid, testicular, prostate and female breast cancers. For 2018, 506,462 new cancer cases were estimated in Brazil. Female breast and prostate were the two main types of cancer in all regions. In the North and Northeast, cervical and stomach cancers stood out. Differences in the I/M ratios between regions were observed and may be related to socioeconomic development and access to health services.

## Resumen

El objetivo de este estudio fue desarrollar una metodología para estimar la incidencia de cáncer en Brasil y sus regiones. A partir de datos de los registros de cáncer de base poblacional (RCBP) y el Sistema de Informaciones de Mortalidad (SIM), se calcularon las tasas anuales de incidencia y mortalidad (I/M), tipo de cáncer, grupo de edad y sexo en cada RCBP. Para estimar las tasas de I/M por región en 2018, se aplicaron modelos multinivel longitudinales de Poisson. Los nuevos casos de cáncer en 2018 se estimaron mediante la aplicación de las tasas I/M que se esperan para el número de muertes corregidas de SIM que habían ocurrido ese año. Las regiones Norte y Nordeste concentraron las más bajas tasas de I/M. Los cánceres de páncreas, pulmón, hígado y esófago tuvieron las más bajas tasas de I/M, mientras que las más altas tasas de I/M se estimaron para los cánceres de tiroides, testículos, próstata y mama femenina. Para 2018, se estimaron 506.462 nuevos casos de cáncer en Brasil. La mama femenina y la próstata representaron técnicas de estimación y configuraron ser los tipos principales de cáncer en todas las regiones. En el Norte y el Nordeste se destacaron los cánceres de cuello uterino y estómago. Se observaron diferencias en las tasas de I/M entre regiones, las cuales pueden estar relacionadas con el desarrollo socioeconómico y el acceso a los servicios de salud.

## Introdução

Conhecer a incidência do câncer é essencial para definir prioridades para os programas de controle, contemplando desde medidas preventivas para fatores de risco até o dimensionamento do sistema de saúde para a assistência ^1^ ^,^ ^2^. Os registros de câncer de base populacional (RCBP) são responsáveis por coletar, processar e analisar as informações sobre os novos casos de câncer ocorridos na população e sua abrangência, fornecendo, assim, subsídios para a vigilância do câncer de forma contínua e sistemática.

No entanto, a abrangência geográfica dos RCBP pode ser limitada e cobrir apenas uma parte da população em uma determinada região ou país. Para as localidades não abrangidas pelos registros, é necessário aplicar outras abordagens a fim de estimar a incidência do câncer e então possibilitar o planejamento e o monitoramento das ações para o controle do câncer.

A razão de incidência e mortalidade (I/M) tem sido amplamente utilizada para estimar a ocorrência do câncer em várias localidades do mundo ^3^ ^,^ ^4^ ^,^ ^5^, incluindo o projeto GLOBOCAN ^6^, liderado pela Agência Internacional para Pesquisa em Câncer (IARC). No Brasil, essa abordagem é adotada para o cálculo das estimativas de câncer realizadas pelo Instituto Nacional de Câncer (INCA) ^7^ ^,^ ^8^. Esse método, descrito por Doll ^9^, pressupõe que a relação entre a incidência e a mortalidade tende a ser geograficamente homogênea com algumas mudanças ao longo do tempo e indica que as razões I/M das áreas abrangidas pelos registros tende a ser a mesma na população regional/nacional, conforme proposto por Black et al. ^10^. Da mesma forma, o inverso da razão I/M, a razão mortalidade/incidência (M/I) é utilizada para avaliar a completude dos RCBP ^11^ e tem sido aplicada como medida aproximada da letalidade bruta ^12^ ^,^ ^13^ ^,^ ^14^ ^,^ ^15^ para o cálculo, também aproximado, da sobrevida em cinco anos, quando não é possível fazer o seguimento apropriado dos casos e conduzir estudos de coorte ^13^. Essa utilidade vem sendo questionada por alguns autores ^15^ ^,^ ^16^, e outros têm indicado seu uso para a avaliação da efetividade de programas de controle de câncer ^17^ ^,^ ^18^.

Ao predizer as razões I/M das regiões não abrangidas por RCBP, no entanto, é importante considerar a variabilidade dessa medida devido a diferenças na estrutura etária, na exposição a fatores de risco, no acesso a ações de rastreamento e detecção precoce, bem como ao tratamento do câncer e outros fatores não conhecidos entre os diferentes RCBP dentro da região. A análise multinível, uma extensão dos modelos lineares mistos generalizados (GLMM), considera a existência de dados agrupados nos quais a variação entre unidades distintas é considerada pela especificação de componentes aleatórios. Nos modelos multiníveis longitudinais, um dos benefícios é permitir que as predições considerem a variabilidade entre os grupos e ao longo do período ^19^. Este trabalho tem como objetivo desenvolver uma metodologia para estimar a incidência de câncer no Brasil e regiões a partir de modelos multiníveis às razões I/M das áreas abrangidas pelos RCBP brasileiros entre 2006 e 2017.

## Métodos

Os dados de incidência e mortalidade foram analisados de acordo com a 10ª revisão da Classificação Internacional de Doenças e Problemas Relacionados à Saúde (CID-10), para 26 grupos de câncer: cabeça e pescoço (C00-C14, C30-C32); esôfago (C15); estômago (C16); colorretal (C18-C20); fígado (C22); vesícula biliar (C23); pâncreas (C25); pulmão (C33-C34); melanoma (C43); mama (C50); vulva (C51); vagina (C52); colo do útero (C53); endométrio (C54); ovário (C56); próstata (C61); testículo (C62); rim (C64); bexiga (C67); cérebro e sistema nervoso central (SNC) (C70-C72); tireoide (C73); linfoma (C81-C85); mieloma (C90); leucemia (C91-C95); outras localizações (D46 e demais códigos entre C00 e C97, excluindo pele não melanoma, C44).

Os casos registrados sem informação de sexo e/ou idade foram redistribuídos de forma proporcional ao local de residência (cidade ou Unidade Federativa conforme abrangência do RCBP) e topografia.

### Dados de incidência

Para os dados de incidência, foram utilizados os microdados dos RCBP, disponibilizados publicamente em novembro de 2021 e sem identificação dos pacientes no sistema BasePopWeb (BPW), disponível em https://www.inca.gov.br/BasePopIncidencias/, para o período de 2007 a 2016.

Os RCBP foram selecionados para o estudo com base nos critérios de qualidade das informações adotados no volume 11 do *Cancer Incidence in Five Continents* ^20^, considerando os seguintes fatores:

(a) Mínimo de três anos na série histórica de 2007 a 2016;

(b) Percentual de casos com diagnóstico por verificação morfológica, citologia, histologia do tumor primário ou da metástase (%VM): > 70%;

(c) Percentual de casos com ausência de informação sobre o método diagnóstico para o diagnóstico do tumor: < 20%;

(d) Percentual de casos identificados somente pela declaração de óbito (%SDO): < 20%; e

(e) Percentual de casos com localizações mal definidas, secundárias e não especificadas: < 20% para as topografias: C76, C77, C78, C79, C80.

### Dados de mortalidade

Os dados de mortalidade utilizados foram obtidos do Sistema de Informações sobre Mortalidade (SIM) no endereço eletrônico (https://ftp.datasus.gov.br/dissemin/publicos/SIM/CID10/DORES) para o período de 2007 a 2016 e para o ano de 2018.

Para o período de 2007 a 2016, os dados de mortalidade foram processados de acordo com a cidade de residência correspondente ao RCBP selecionado. Já para o ano de 2018, os dados foram processados segundo região de residência.

Conforme método proposto por Mathers et al. ^21^ e aplicado em estudos recentes ^22^ ^,^ ^23^, 50% dos óbitos classificados como mal definidos foram redistribuídos proporcionalmente para cada tipo de câncer por ano do óbito, local de residência, sexo e faixa etária em anos (0-19, 20-29, 30-39, 40-49, 50-59, 60-69, 70 ou mais), excluindo-se as causas externas definidas (CID-10 capítulo 20, códigos V01-Y98). Foram considerados como causas mal definidas os seguintes códigos da CID-10: R00-R99, I46.1, I46.9, I95.9, J96.0 e J96.9.

Da mesma forma, os códigos-lixo de câncer foram distribuídos proporcionalmente segundo ano do óbito, local de residência, sexo e faixa etária em anos (0-19, 20-29, 30-39, 40-49, 50-59, 60-69, 70 ou mais) aos códigos-alvo específicos de câncer conforme Quadro 1.

**Quadro 1:** Esquema de recodificação na base de mortalidade dos códigos-lixo e respectivos códigos-alvo.

CÓDIGOS-LIXO	CÓDIGOS-ALVO
ESPECÍFICOS	
C26.0	C18-C21
C55	C53-C54
C76.0	C00-C14; C30-C32
INESPECÍFICOS	
C26.8; C26.9	C15-C25
C57.8; C57.9	C51-C54; C56-C57.4; C57.7
C76.2	C15-C25; C48; C49.4
C76.3	C19-C20; C49.5; C51-C54; C56-C57.4; C57.7; C67
C78-C79	C16; C18; C22; C25; C33-C34; C56; C64
C80 *	C16; C18; C22; C25; C33-C34; C56; C64

* Foram distribuídos apenas o que excedeu 5% do total de óbitos por câncer.

### Cálculo das razões incidência-mortalidade (I/M)

Considerando apenas os dados dos RCBP selecionados, foram calculadas as razões I/M nas áreas cobertas pelos RCBP entre 2007 e 2016. Esse cálculo foi realizado para cada topografia, sexo e faixa etária em anos (0-29, 30-49, 50-59, 60-69, 70 ou mais), dividindo o número anual de casos novos registrados no RCBP pelo número corrigido de óbitos no mesmo ano.

Para as predições das razões I/M nas cinco regiões e para cada uma das 27 topografias de câncer, foram aplicados modelos multiníveis de Poisson, tendo o ano como unidade de primeiro nível e o RCBP como unidade de segundo nível, com intercepto aleatório, estratificados por sexo e faixa etária. Em razão das diferentes distribuições dos tipos de câncer por faixa etária, foi necessário, para algumas topografias, agrupar em diferentes faixas: (a) cabeça e pescoço, esôfago, colorretal, fígado, pâncreas, pulmão, melanoma, corpo do útero, próstata, bexiga, mieloma e primário desconhecido (0-49, 50-59, 60-69, 70 anos ou mais); (b) vesícula biliar, vulva, tireoide (0-59, 60-69, 70 anos ou mais); (c) vagina (0-69, 70 anos ou mais); e (d) testículo (0-29, 30-49, 50 anos ou mais).

A variável resposta foi a razão I/M por ano e RCBP. O intercepto aleatório foi incluído de forma a considerar a influência de características não mensuradas de cada RCBP. Embora já tenham sido selecionados para o estudo apenas os RCBP com três anos no período para o conjunto dos dados, foram selecionadas para a entrada no modelo estratificado as informações daqueles que tiveram, ao menos, dois anos na série nos estratos de topografia, idade e sexo selecionados. Considerando que há grande variação entre os RCBP em relação às informações registradas ao longo do tempo, o inverso da variância da razão I/M de cada RCBP ao longo dos anos, por topografia, sexo e faixa etária foi utilizado como peso.

Dessa forma, a equação do modelo estimado de forma estratificada por sexo e faixa etária para cada topografia foi a seguinte:

$log\left(\mu_{jt}\right)=\beta_{0}+\beta_{1}{ano}_{t}+\gamma_{r}{região}_{rj}+u_{j}$

Em que:

*β*_*0*_ *+ u*_*j*_ é o intercepto aleatório do RCBP;

*β*_*1*_ é o efeito fixo do tempo;

*γ*_*r*_ *= (γ*_*1*_ *, ..., γ*_*R-1*_ *)* é um vetor de coeficientes para as variáveis indicadoras da região do RCBP;

*região*_*rj*_ com *r = 1, ..., R-1*;

*R* é o número de regiões.

Para testar a significância do efeito dos RCBP, foram conduzidos testes de razão de verossimilhança comparando o modelo multinível com e sem o efeito aleatório dos RCBP. Para estimar as razões I/M para cada topografia em 2018 por sexo e faixa etária nas regiões brasileiras por esses modelos, a média das razões I/M foi modelada por uma combinação de efeitos aleatórios e fixos utilizando o comando *margins* no Stata, versão 17.0 (https://www.stata.com).

### Estimativa de incidência

Para o cálculo da estimativa de casos novos em 2018, as razões I/M preditas para 2018 pelos modelos foram aplicadas aos óbitos corrigidos em 2018 nos mesmos estratos. Quando as razões I/M estimadas eram menores que 1, foram definidas como 1, de forma a evitar a subestimativa dos casos novos.

As razões I/M para o Brasil foram calculadas como a razão entre a soma das incidências estimadas e o número corrigido de óbitos em 2018 nas regiões por topografia e para todos os cânceres exceto pele não melanoma.

## Resultados

Foram analisados 29 RCBP, cujos dados referentes ao período de diagnóstico de 2007 a 2016 estavam disponíveis no momento da coleta. Desses, 19 foram incluídos nas análises ( Material Suplementar - Tabelas S1 e S2 https://cadernos.ensp.fiocruz.br/static//arquivo/suppl-e00131623_7126.pdf).

As regiões Norte e Nordeste apresentaram as menores razões I/M projetadas para 2018 para a maior parte das topografias e para o conjunto delas, excluindo pele não melanoma: 1,87 e 2,04, respectivamente. Por outro lado, as regiões Sul (2,28) e Sudeste (2,3) tiveram as maiores razões I/M (Tabela 1).

**Tabela 1:** Razões de incidência e mortalidade (I/M) estimadas para 2018 no Brasil e regiões brasileiras por tipo de câncer, ambos os sexos.

Tipo de câncer	Razões I/M					
	Brasil	Norte	Nordeste	Sudeste	Sul	Centro-oeste
Bexiga	2,40	1,63	1,92	2,52	2,74	2,00
Cabeça e pescoço	1,84	1,48	1,69	1,91	1,96	1,73
Cérebro e SNC	1,36	1,37	1,39	1,24	1,57	1,49
Colo do útero	2,17	2,24	2,06	2,00	2,79	2,09
Colorretal	2,07	1,62	1,81	2,03	2,49	2,11
Corpo do útero	3,40	2,15	3,55	3,26	4,06	3,33
Esôfago	1,27	1,39	1,20	1,24	1,34	1,44
Estômago	1,38	1,35	1,34	1,33	1,52	1,53
Fígado	1,11	1,23	1,09	1,02	1,34	1,11
Leucemia	1,71	1,69	2,04	1,41	2,04	1,70
Linfoma	2,82	2,05	2,47	2,78	3,44	2,78
Mama	4,27	3,40	3,62	4,62	4,40	4,05
Melanoma	3,72	1,90	2,80	3,88	4,18	3,02
Mieloma	1,57	1,51	1,57	1,51	1,75	1,64
Ovário	1,83	1,86	2,09	1,69	1,83	1,92
Pâncreas	1,07	1,11	1,06	1,04	1,13	1,15
Próstata	4,61	3,27	3,81	5,45	4,51	3,99
Pulmão	1,10	1,12	1,10	1,07	1,14	1,12
Rim	2,90	2,23	2,32	3,04	3,24	2,71
Testículo	5,28	2,15	2,02	6,42	6,45	2,82
Tireoide	24,69	10,79	16,27	39,28	14,02	10,06
Vagina	1,69	2,14	1,27	1,93	1,00	1,53
Vesícula biliar	1,39	1,38	1,41	1,35	1,39	1,58
Vulva	1,87	1,66	1,92	1,97	1,70	1,65
Outras localizações	2,15	2,01	2,11	2,15	2,13	2,48
Todos (exceto C44)	2,20	1,87	2,04	2,30	2,28	2,15

SNC: sistema nervoso central.

Legenda para a escala de cores das razões I/M por tipo de câncer entre as regiões:

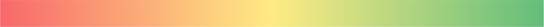

Menor razão I/M Maior razão I/M

Os cânceres de pâncreas (I/M = 1,07), pulmão (I/M = 1,1), fígado (I/M = 1,11) e esôfago (I/M = 1,27) tiveram as menores razões I/M estimadas para 2018 no Brasil em ambos os sexos. Já as maiores razões I/M foram estimadas para câncer de tireoide (I/M = 24,69), testículo (I/M = 5,28), próstata (I/M = 4,61) e mama feminina (I/M = 4,27). Para as topografias com menores razões I/M, a variabilidade entre as regiões foi menor do que para aquelas com maiores razões I/M (Tabela 1).

Para o conjunto dos cânceres excluindo pele não melanoma e para a maior parte dos tumores comuns aos dois sexos, as mulheres tiveram razões I/M mais elevadas que os homens, padrão que se repetiu nas regiões. A maior diferença entre as razões nos dois sexos foi para o câncer de tireoide (I/M homens = 9,15; I/M mulheres = 32,19), enquanto a menor foi para o câncer de estômago (I/M homens = 1,35; I/M mulheres = 1,43) (Tabela 2).

**Tabela 2:** Razões de incidência e mortalidade (I/M) estimadas para 2018 no Brasil e regiões brasileiras por tipo de câncer, segundo sexo.

Tipo de câncer	Razões I/M											
	Homens						Mulheres					
	Brasil	Norte	Nordeste	Sudeste	Sul	Centro-oeste	Brasil	Norte	Nordeste	Sudeste	Sul	Centro-oeste
Bexiga	2,50	1,69	1,92	2,60	2,94	2,13	2,18	1,52	1,93	2,34	2,27	1,76
Cabeça e pescoço	1,75	1,41	1,60	1,83	1,87	1,56	2,18	1,70	1,97	2,26	2,39	2,45
Cérebro e SNC	1,42	1,39	1,48	1,23	1,71	1,57	1,31	1,34	1,30	1,25	1,41	1,39
Colo do útero							2,17	2,24	2,06	2,00	2,79	2,09
Colorretal	2,15	1,76	1,94	2,09	2,55	2,07	2,00	1,49	1,71	1,97	2,43	2,15
Corpo do útero							3,40	2,15	3,55	3,26	4,06	3,33
Esôfago	1,24	1,39	1,16	1,20	1,34	1,34	1,41	1,38	1,35	1,43	1,36	1,78
Estômago	1,35	1,37	1,31	1,30	1,49	1,46	1,43	1,32	1,39	1,38	1,57	1,65
Fígado	1,12	1,20	1,11	1,01	1,37	1,07	1,10	1,27	1,07	1,02	1,28	1,17
Leucemia	1,64	1,67	1,97	1,35	1,89	1,63	1,81	1,71	2,12	1,48	2,21	1,80
Linfoma	2,76	2,04	2,46	2,65	3,47	2,72	2,88	2,06	2,48	2,93	3,40	2,86
Mama							4,27	3,40	3,62	4,62	4,40	4,05
Melanoma	3,53	1,91	2,67	3,58	4,11	2,44	3,97	1,89	2,96	4,30	4,31	3,54
Mieloma	1,52	1,38	1,56	1,44	1,72	1,50	1,64	1,70	1,57	1,59	1,78	1,83
Ovário							1,83	1,86	2,09	1,69	1,83	1,92
Pâncreas	1,03	1,04	1,01	1,01	1,07	1,06	1,12	1,18	1,11	1,07	1,19	1,26
Próstata	4,61	3,27	3,81	5,45	4,51	3,99						
Pulmão	1,05	1,11	1,06	1,03	1,07	1,05	1,16	1,13	1,15	1,11	1,25	1,20
Rim	2,92	2,37	2,50	2,89	3,42	2,86	2,87	2,05	2,06	3,33	2,93	2,43
Testículo	5,28	2,15	2,02	6,42	6,45	2,82						
Tireoide	9,15	2,01	5,42	14,10	7,36	4,09	32,19	15,64	20,80	52,07	17,20	13,36
Vagina							1,69	2,14	1,27	1,93	1,00	1,53
Vesícula biliar	1,49	1,32	1,62	1,43	1,43	1,61	1,36	1,42	1,34	1,32	1,37	1,57
Vulva							1,87	1,66	1,92	1,97	1,70	1,65
Outras localizações	1,95	1,86	1,91	1,93	1,90	2,37	2,36	2,17	2,29	2,37	2,37	2,61
Todos (exceto C44)	2,04	1,73	1,92	2,10	2,13	1,98	2,38	2,03	2,16	2,51	2,45	2,35

SNC: sistema nervoso central.

Legenda para a escala de cores das razões I/M por tipo de câncer entre as regiões:

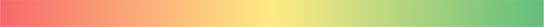

Menor razão I/M Maior razão I/M

Para o ano de 2018, foram estimados 506.462 novos casos de câncer exceto pele não melanoma no Brasil, sendo que a Região Sudeste concentrou quase metade dos casos (n = 276.656; 49,65%). Mama feminina, próstata, colorretal, pulmão e cabeça e pescoço são os cinco tipos mais comuns em ambos os sexos e compreendem pouco mais da metade de todos os tipos de câncer estimados para 2018 (Tabela 3).

**Tabela 3:** Número estimado de casos novos de câncer em 2018, por tipo de câncer, no Brasil e regiões brasileiras.

Tipo de câncer/Sexo	Brasil		Norte		Nordeste		Sudeste		Sul		Centro-oeste	
	Casos novos	%	Casos novos	%	Casos novos	%	Casos novos	%	Casos novos	%	Casos novos	%
Bexiga												
Homens	7.806	3,18	179	1,65	992	1,99	4.245	3,56	1.971	3,92	419	2,67
Mulheres	3.272	1,25	94	0,81	524	0,97	1.842	1,41	638	1,32	173	1,05
Ambos	11.078	2,19	274	1,21	1.516	1,46	6.087	2,44	2.609	2,65	593	1,84
Cabeça e pescoço												
Homens	21.380	8,70	785	7,20	4.248	8,55	11.057	9,29	4.043	8,05	1.247	7,94
Mulheres	6.653	2,55	304	2,61	1.572	2,91	3.194	2,45	1.116	2,31	466	2,82
Ambos	28.032	5,53	1.089	4,83	5.820	5,61	14.252	5,71	5.159	5,24	1.714	5,32
Cérebro e SNC												
Homens	6.192	2,52	367	3,37	1.492	3,00	2.359	1,98	1.437	2,86	537	3,42
Mulheres	5.180	1,99	294	2,52	1.212	2,24	2.210	1,70	1.073	2,22	391	2,37
Ambos	11.372	2,25	662	2,93	2.704	2,61	4.568	1,83	2.510	2,55	928	2,88
Colo do útero												
Homens	-	-	-	-	-	-	-	-	-	-	-	-
Mulheres	18.534	7,10	2.394	20,52	5.281	9,77	5.897	4,53	3.551	7,35	1.410	8,53
Colorretal												
Homens	25.731	10,48	733	6,73	3.358	6,76	13.933	11,70	6.008	11,96	1.700	10,82
Mulheres	24.986	9,58	647	5,55	3.759	6,95	13.346	10,24	5.508	11,40	1.725	10,44
Ambos	50.717	10,01	1.380	6,12	7.117	6,86	27.279	10,94	11.516	11,69	3.425	10,63
Corpo do útero												
Homens	-	-	-	-	-	-	-	-	-	-	-	-
Mulheres	8.292	3,18	173	1,49	1.582	2,93	4.615	3,54	1.519	3,14	402	2,43
Esôfago												
Homens	8.829	3,60	321	2,95	1.706	3,43	4.134	3,47	2.131	4,24	537	3,42
Mulheres	2.839	1,09	85	0,73	626	1,16	1.199	0,92	699	1,45	231	1,40
Ambos	11.669	2,30	406	1,80	2.332	2,25	5.333	2,14	2.830	2,87	768	2,38
Estômago												
Homens	14.032	5,71	1.263	11,60	3.216	6,47	6.025	5,06	2.705	5,39	823	5,24
Mulheres	8.533	3,27	594	5,09	2.156	3,99	3.709	2,85	1.495	3,10	578	3,50
Ambos	22.565	4,46	1.857	8,23	5.373	5,18	9.734	3,90	4.201	4,26	1.401	4,35
Fígado												
Homens	7.642	3,11	575	5,28	1.858	3,74	3.019	2,54	1.749	3,48	440	2,80
Mulheres	5.357	2,05	348	2,98	1.543	2,85	2.167	1,66	975	2,02	325	1,96
Ambos	12.999	2,57	923	4,09	3.401	3,28	5.186	2,08	2.724	2,77	765	2,37
Leucemia												
Homens	6.655	2,71	444	4,07	1.891	3,81	2.440	2,05	1.369	2,73	510	3,25
Mulheres	6.175	2,37	360	3,09	1.730	3,20	2.233	1,71	1.429	2,96	423	2,56
Ambos	12.830	2,53	804	3,56	3.621	3,49	4.674	1,87	2.798	2,84	933	2,89
Linfoma												
Homens	7.500	3,05	253	2,32	1.387	2,79	3.449	2,90	1.971	3,92	441	2,81
Mulheres	6.641	2,55	192	1,64	1.239	2,29	3.356	2,58	1.490	3,08	364	2,20
Ambos	14.141	2,79	445	1,97	2.626	2,53	6.805	2,73	3.460	3,51	805	2,50
Mama												
Homens	-	-	-	-	-	-	-	-	-	-	-	-
Mulheres	77.278	29,62	2.752	23,59	14.246	26,35	41.839	32,11	13.492	27,93	4.950	29,95
Melanoma												
Homens	3.803	1,55	65	0,59	403	0,81	1.588	1,33	1.613	3,21	135	0,86
Mulheres	3.071	1,18	31	0,27	371	0,69	1.353	1,04	1.100,00	2,28	215	1,30
Ambos	6.873	1,36	96	0,43	773	0,75	2.941	1,18	2.713	2,75	350	1,09
Mieloma												
Homens	2.895	1,18	104	0,95	635	1,28	1.314	1,10	596	1,19	245	1,56
Mulheres	2.785	1,07	84	0,72	631	1,17	1.362	1,05	496	1,03	212	1,28
Ambos	5.680	1,12	187	0,83	1.267	1,22	2.676	1,07	1.093	1,11	457	1,42
Ovário												
Homens	-	-	-	-	-	-	-	-	-	-	-	-
Mulheres	8.108	3,11	393	3,37	2.130	3,94	3.655	2,81	1.365	2,82	566	3,42
Pâncreas												
Homens	6.215	2,53	244	2,24	1.219	2,45	2.954	2,48	1.378	2,74	420	2,67
Mulheres	6.926	2,66	283	2,43	1.320	2,44	3.449	2,65	1.456	3,01	419	2,53
Ambos	13.141	2,59	526	2,33	2.539	2,45	6.403	2,57	2.834	2,88	839	2,60
Próstata												
Homens	74.207	30,21	3.073	28,21	17.290	34,79	37.301	31,32	12.111	24,12	4.431	28,22
Mulheres	-	-	-	-	-	-	-	-	-	-	-	-
Pulmão												
Homens	18.550	7,55	955	8,77	3.566	7,17	8.383	7,04	4.426	8,81	1.220	7,77
Mulheres	15.327	5,88	634	5,44	3.309	6,12	6.759	5,19	3.646	7,55	980	5,93
Ambos	33.877	6,69	1.590	7,05	6.875	6,62	15.141	6,07	8.071	8,19	2.200	6,83
Rim												
Homens	7.209	2,94	262	2,41	994	2,00	3.631	3,05	1.843	3,67	479	3,05
Mulheres	4.104	1,57	169	1,45	549	1,01	2.226	1,71	949	1,97	210	1,27
Ambos	11.312	2,23	431	1,91	1.542	1,49	5.857	2,35	2.792	2,83	689	2,14
Testículo												
Homens	2.230	0,91	79	0,72	106	0,21	1.166	0,98	804	1,60	76	0,49
Mulheres	-	-	-	-	-	-	-	-	-	-	-	-
Tireoide												
Homens	2.575	1,05	38	0,35	385	0,77	1.715	1,44	340	0,68	96	0,61
Mulheres	18.777	7,20	538	4,61	3.536	6,54	12.468	9,57	1.667	3,45	568	3,44
Ambos	21.352	4,22	576	2,55	3.921	3,78	14.183	5,69	2.007	2,04	664	2,06
Vagina												
Homens	-	-	-	-	-	-	-	-	-	-	-	-
Mulheres	229	0,09	34	0,29	29	0,05	132	0,10	19	0,04	16	0,09
Vesícula biliar												
Homens	536	0,22	45	0,42	177	0,36	210	0,18	72	0,14	32	0,20
Mulheres	1.293	0,50	80	0,69	397	0,73	524	0,40	192	0,40	99	0,60
Ambos	1.829	0,36	126	0,56	575	0,55	734	0,29	264	0,27	131	0,41
Vulva												
Homens	-	-	-	-	-	-	-	-	-	-	-	-
Mulheres	811	0,31	19	0,16	131	0,24	452	0,35	160	0,33	49	0,30
Outras localizações												
Homens	21.615	8,80	1.107	10,17	4.780	9,62	10.158	8,53	3.655	7,28	1.915	12,19
Mulheres	25.689	9,85	1.161	9,95	6.191	11,45	12.307	9,45	4.274	8,85	1.755	10,62
Ambos	47.305	9,34	2.268	10,06	10.971	10,57	22.465	9,01	7.929	8,05	3.670	11,39
Todos (exceto C44)												
Homens	245.603	100,00	10.893	100,00	49.703	100,00	119.083	100,00	50.222	100,00	15.703	100,00
Mulheres	260.859	100,00	11.664	100,00	54.065	100,00	130.294	100,00	48.308	100,00	16.527	100,00
Ambos	506.462	100,00	22.557	100,00	103.768	100,00	249.377	100,00	98.530	100,00	32.230	100,00

SNC: sistema nervoso central.

Entre os homens, o câncer de próstata foi quase três vezes mais frequente (n = 74.207; 30,31%) do que o colorretal (n = 25.731; 10,48%), o segundo de maior incidência. Os cânceres de cabeça e pescoço (n = 21.380; 8,7%), pulmão (n = 18.550; 7,55%) e estômago (n = 14.032; 5,71%) ocuparam, respectivamente, da terceira à quinta posição em incidência nesse grupo. Em todas as regiões, esses foram os cinco principais tipos de câncer nos homens, com algumas variações na ordem das topografias para o Norte, Nordeste e Sul. Na Região Norte, o câncer de estômago foi o segundo mais incidente (n = 1.263; 11,6%) (Tabela 3).

Na população feminina, salienta-se o fato de o câncer de mama (n = 77.278; 29,62%) ser o mais frequente, seguido pelo câncer colorretal (n = 24.986; 9,58%). Em seguida, o terceiro ao quinto mais incidentes no país foram: câncer de tireoide (n = 18,777; 7,2%); colo do útero (n = 18.534; 7,1%); e pulmão (n = 15.327; 5,88%). Merece destaque o número de casos estimados de câncer do colo do útero para a Região Norte (n = 2.394; 20,52%), apenas três pontos percentuais abaixo do câncer de mama (Tabela 3).

## Discussão

Este estudo identificou grande variação nas razões I/M estimadas entre as regiões brasileiras, sendo as regiões Norte e Nordeste aquelas com as menores razões I/M estimadas. Para os tipos de câncer mais incidentes, também foram observadas diferenças regionais com maior peso para o câncer do colo do útero e de estômago na Região Norte. Essas variações indicam as desigualdades de acesso aos serviços de saúde e ações de prevenção e controle do câncer. As regiões Norte e Nordeste são as regiões brasileiras com menores índices de desenvolvimento humano, maiores índices de desigualdade e pior acesso a serviços de saúde ^24^. Esses resultados convergem para o que os estudos internacionais têm apontado, ao identificar menores razões I/M para localidades onde o sistema de saúde foi pior classificado ou tinha ações de prevenção e controle implementadas em estágio menos avançado ^17^ ^,^ ^18^. Um estudo recente, que avaliou a utilização dos serviços de saúde no Brasil a partir de dados da *Pesquisa Nacional de Saúde* (PNS) de 2013 e 2019, indicou que persistem necessidades em saúde não atendidas entre os residentes das regiões menos desenvolvidas, independentemente do rendimento ou porte de plano de saúde ^25^. Assim, o acesso às ações de controle do câncer pode ser, para além de fatores socioeconômicos, influenciado pela capilaridade e organização da rede de atenção e pela oferta dos serviços relacionados ^26^.

Iniquidades regionais de acesso às ações de rastreamento e detecção precoce indicam que existe menor oferta tanto de equipamentos como de recursos humanos especializados nas regiões Norte e Nordeste ^27^. Tais diferenças são associadas a menores coberturas dos exames, sendo seu impacto nessas regiões maior do que as desigualdades econômicas ^28^. Da mesma forma, de acordo com Silva et al. ^29^, o número de hospitais habilitados na Região Norte é o menor do país, sendo os pacientes dessa região, assim como do Centro-oeste, aqueles que precisam percorrer maiores distâncias para obter tratamento oncológico ^30^, tendo maiores chances de atraso no início do tratamento ^31^ ^,^ ^32^.

Assim como para as disparidades entre as regiões, é possível que as diferenças observadas nas razões I/M de homens e mulheres também estejam relacionadas à menor utilização dos serviços de saúde pelos homens. De acordo com resultados da PNS de 2019 ^25^, a procura por serviços de saúde para a prevenção e por motivo de doença ou problema de saúde foram, respectivamente, quase 70% e 50% maiores entre as mulheres em comparação aos homens. Tais comportamentos possivelmente refletem o uso dos serviços de saúde para o tratamento do câncer: um estudo com dados do Painel-Oncologia do período de 2013 a 2019 indicou que a chance de iniciar o tratamento em até 60 dias após o diagnóstico foi maior entre as mulheres do que entre os homens em todas as regiões brasileiras, exceto a Norte ^33^.

O câncer de tireoide foi aquele com maior diferença nas razões I/M por sexo e região. Nas mulheres, a razão I/M foi 3,5 vezes maior que nos homens. Contudo, é provável que essa diferença seja, em parte, atribuída ao maior acesso das mulheres aos procedimentos diagnósticos, levando à detecção de pequenos tumores do tipo papilar, tipo mais indolente desse câncer ^34^.

Considerando a tendência de queda na mortalidade por câncer de mama e próstata desde meados dos anos 1990 e da primeira década dos anos 2000 ^22^, respectivamente, as maiores razões I/M observadas para o Sudeste e Sul, para os cânceres de próstata e mama, que são dos mais curáveis, podem ser indicadoras de melhor acesso ao rastreamento com detecção de tumores indolentes, especialmente no caso da próstata. É possível também que esse fato reflita a melhora dos procedimentos terapêuticos.

Para os tipos de câncer mais agressivos, como pâncreas, pulmão, fígado e esôfago, as diferenças nas razões I/M entre as regiões e entre homens e mulheres foram consideravelmente menos acentuadas, pois são cânceres cujos avanços terapêuticos atualmente disponíveis ainda são limitados e não modificam a evolução clínica da maioria dos casos.

A razão I/M é um importante componente da vigilância do câncer, apresentando uma perspectiva sobre a relação entre a incidência e a mortalidade. Metodologias que exploram a relação entre a incidência e a mortalidade das localidades abrangidas pelos RCBP são amplamente utilizadas para estimar a incidência de câncer em escala nacional ^6^ ^,^ ^9^ ^,^ ^10^ ^,^ ^35^ ^,^ ^36^ ^,^ ^37^ ^,^ ^38^ ^,^ ^39^ ^,^ ^40^ ^,^ ^41^. A razão I/M é utilizada para o cálculo de estimativas de incidência de câncer desde os anos 1960 ^9^ e seu emprego para essa finalidade tem sido validado, indicando que esse método fornece estimativas confiáveis para a maior parte dos cânceres ^42^ ^,^ ^43^. No Brasil, as estimativas oficiais de incidência de câncer publicadas pelo INCA utilizam a razão I/M em sua metodologia desde o ano 2000 ^44^.

A razão M/I, ou seja, o inverso da razão I/M, já foi aplicada para a comparação da carga do câncer entre diferentes localidades ^45^. Para o Brasil, não identificamos estudos que comparem o comportamento dessa medida entre diferentes regiões e para diversos tipos de câncer. Alguns estudos aplicaram essa medida para avaliar desigualdades socioeconômicas ^45^, de gênero ^46^, de raça/cor da pele ^47^, acesso aos serviços de saúde e para a avaliação da efetividade de programas de controle de câncer, principalmente para as ações de rastreamento ^17^ ^,^ ^48^. No entanto, é preciso considerar que é uma medida sensível a mudanças na incidência e na mortalidade, bem como à cobertura dos registros de incidência e à qualidade das informações de mortalidade.

Neste estudo, quando a razão I/M foi menor do que 1, substituímos o valor por 1 para evitar a subestimação dos casos incidentes. Essa situação ocorreu especialmente para tumores de maior letalidade. Essa correção impactou em 1,1% dos casos (5.626) sendo 66% dessa diferença (3.714 casos) referente aos cânceres de pulmão, fígado e pâncreas.

A razão I/M pode ter limitações quando estimada para tipos de câncer cujas políticas de rastreamento e detecção precoce estão sujeitas a mudanças. Para tumores que são passíveis de detecção em estágios pré-malignos (lesão precursora), como o câncer do colo do útero e colorretal, a implantação de ações de rastreamento e detecção precoce pode implicar no aumento da incidência e, por consequência, maiores razões I/M no período inicial das ações, dado que o impacto na mortalidade é posterior. Da mesma forma, devem ser consideradas mudanças na incidência devido ao sobrediagnóstico, como é o caso do câncer de mama, próstata, tireoide, rim e melanoma ^49^ ^,^ ^50^. Nessas situações, a incidência pode aumentar devido ao maior diagnóstico de tumores indolentes, que não produzem incrementos na mortalidade ao longo do tempo.

Para os casos novos de câncer estimados por este estudo, foram observados padrões de incidência que refletem aspectos demográficos, exposições a fatores de risco e acesso aos serviços de saúde nas regiões brasileiras. Enquanto nas regiões Sudeste e Sul prevaleceram tumores associados ao envelhecimento, no Norte, destacou-se a proporção de casos novos de câncer colo do útero e estômago, ambos resultantes do baixo desenvolvimento socioeconômico, associados a agentes infecciosos ^51^ e ao baixo acesso aos serviços de saúde ^52^.

A comparação entre os achados deste estudo e estimativas desenvolvidas pela IARC ^41^ e pelo INCA ^8^ não pode ser feita de forma direta. A série temporal das informações e o número de RCBP divergem entre as estimativas para 2018: o INCA incluiu RCBP com informações referentes ao período de 2001-2014; porém, sem especificar a seleção dos registros de melhor qualidade ^8^. Já a IARC ^41^ utilizou informações de 2003 a 2007, selecionadas a partir de critérios de qualidade ^53^. Neste estudo, utilizamos RCBP selecionados a partir dos critérios de qualidade adotados pela IARC ^20^, com informações para o período de 2007 a 2016 ( Material Suplementar - Tabelas S1 e S2 https://cadernos.ensp.fiocruz.br/static//arquivo/suppl-e00131623_7126.pdf).

Apesar de serem resultantes de diferentes metodologias, uma comparação entre as razões I/M estimadas por este estudo e as calculadas pelos dados estimados pelo GLOBOCAN da IARC para os tipos de câncer mais incidentes no Brasil em 2018 ^54^ (Tabela 4) mostrou poucas diferenças, reforçando a robustez dos dados deste estudo. Assim como nesta pesquisa, o GLOBOCAN aplica metodologia para a redistribuição de causas mal definidas e não específicas de óbito para melhorar a qualidade dos dados ^41^.

**Tabela 4:** Comparação entre os resultados (número de casos novos e razões de incidência e mortalidade - I/M) deste estudo e as estimativas do GLOBOCAN ^55^ e do Instituto Nacional de Câncer (INCA) ^8^ para 2018 por tipos selecionados de câncer no Brasil.

Tipo de câncer	Este estudo			GLOBOCAN			INCA
	Casos estimados	Óbitos registrados no SIM e corrigidos	Razão I/M	Casos estimados	Óbitos estimados	Razão I/M	Casos estimados
Mama feminina	77.278	18.085	4,27	85.620	18.442	4,64	59.700
Próstata	74.207	16.111	4,61	84.992	16.730	5,08	68.220
Colorretal	50.717	24.449	2,07	49.096	23.868	2,06	36.360
Pulmão *	33.877	30.904	1,10	34.511	31.856	1,08	31.270
Estômago	22.565	16.850	1,34	20.927	15.796	1,32	21.290
Tireoide	21.352	865	24,69	21.470	1.009	21,28	9.610
Colo do útero	18.534	8.536	2,17	16.298	8.079	2,02	16.370
Todos (exceto C44)	506.462	229.690	2,20	527.264	241.134	2,19	417.010

SIM: Sistema de Informações sobre Mortalidade.

* Traqueia, brônquios e pulmão.

Uma vez que a estimativa de incidência do INCA para 2018 não indica o número de óbitos estimados para 2018, não foi possível comparar as razões I/M. Por outro lado, é possível observar maior aproximação dos resultados do presente estudo aos apresentados pelo GLOBOCAN para 2018. A falta de redistribuição das causas mal definidas e não específicas de óbito pode ter impactado os números estimados pelo INCA para 2018 (Tabela 4).

As razões I/M estimadas, aplicadas à mortalidade corrigida para 2019 permitiram o cálculo dos casos estimados para 2019. Desses (n = 517.926), foi descontada a cobertura da saúde suplementar daquele ano no país (24,2%), o que corresponde a uma estimativa de 392.588 casos novos sem plano de saúde em 2019. Considerando o número de casos diagnosticados no Sistema Único de Saúde (SUS), exceto pele não melanoma, constantes do Painel-Oncologia (n = 397.208), foi observado que as estimativas aqui produzidas são capazes de refletir com acurácia os casos incidentes encaminhados na rede do SUS. O Painel-Oncologia é uma ferramenta do Ministério da Saúde para o monitoramento do intervalo de tempo até o diagnóstico dos casos diagnosticados e tratados no SUS ^55^. O ano de 2019 foi definido para essa análise por limitações dos sistemas de informação que alimentam a base do Painel-Oncologia, para as quais o registro de CID-10 de diagnóstico passou a ser obrigatório a partir de meados de 2018 ^55^.

Embora a qualidade dos dados de mortalidade no Brasil tenha melhorado nas últimas décadas, o percentual de causas mal definidas ainda é considerado elevado para os padrões internacionais. Entre as regiões brasileiras, persistem diferenças na proporção de óbitos registrados como mal definidos, sendo a qualidade melhor nas regiões com maior nível socioeconômico, Sul e Sudeste, e pior no Norte e Nordeste ^56^. Considerando que o câncer é menos encontrado entre os óbitos mal definidos do que entre os bem-definidos ^57^, este estudo redistribuiu proporcionalmente 50% dos óbitos registrados com causa básica conforme metodologia aplicada anteriormente ^22^. Além disso, para melhorar a qualidade dos dados de mortalidade específica por câncer, os óbitos registrados com códigos de câncer não específicos (os códigos-lixo) foram redistribuídos para códigos específicos, de acordo com critérios clínicos.

Para lidar com as limitações na falta de cobertura e continuidade das informações dos RCBP, foram adotados critérios para a seleção dos RCBP. A classificação dos casos e a seleção dos RCBP de acordo com critérios de qualidade internacionalmente aplicados permite a comparabilidade e a validade das informações ^58^. Além disso, os dados utilizados para as estimativas compreenderam um período de dez anos e um tempo mínimo de contribuição dos registros ao longo da série foi considerado.

Os modelos de efeitos aleatórios para a estimativa da razão I/M mostraram-se adequados, permitindo considerar a variação dos efeitos aleatórios dos RCBP que não seriam captados pelos modelos tradicionais de efeitos fixos. A introdução do efeito aleatório no RCBP permitiu que a respectiva reta de regressão pudesse ter um intercepto diferente para cada registro, sendo considerado no efeito médio para a região em que o RCBP estava localizado. Da mesma forma, os efeitos do tempo permitiram que as curvas fossem ajustadas com uma trajetória individual ao longo do tempo, quantificando a variação da razão I/M dos RCBP nas regiões. Uma outra característica desses modelos é que eles comportam medidas desbalanceadas no tempo e mensurações incompletas como as diferenças de disponibilidade das informações dos registros dentro de uma região ^59^.

Devido à falta de RCBP com abrangência regional, é necessário utilizar métodos indiretos para o cálculo das estimativas de incidência da doença. Esses métodos vêm sendo aperfeiçoados com o aprimoramento dos modelos estatísticos empregados. Entretanto, a acurácia das estimativas produzidas a partir deles varia para cada tipo de câncer ^60^ e é afetada não só pela cobertura e qualidade dos RCBP como pela qualidade das informações de mortalidade das regiões onde a incidência será estimada.

Apesar das limitações apontadas, a metodologia utilizada neste estudo se mostrou factível, permitindo o cálculo da estimativa a curto prazo da razão I/M e do número de casos incidentes a partir de uma abordagem regional e longitudinal. Essas estimativas são importantes para orientar a alocação eficiente de recursos para o planejamento de políticas de saúde e diminuição das desigualdades de acesso aos serviços de saúde entre as regiões brasileiras. Para garantir que as estimativas sejam realizadas de forma mais acurada, permitindo o planejamento de recursos de forma a atender às diferentes necessidades das ações de controle de câncer nas regiões brasileiras, é importante que, no cálculo de estimativas de incidência, sejam empregados dados qualificados e que os dados de mortalidade sejam corrigidos para causas mal definidas e códigos não específicos. Ao mesmo tempo, esforços devem ser feitos para garantir a continuidade, a cobertura e a qualidade da informação dos registros de câncer de base populacional.
