# Supplementary material for: Estimation of cancer incidence in Brazil and its regions in 2018: methodological aspects
Source: Cad Saude Publica. 2024 Jul 29;40(6):e00131623. [Article in Portuguese] doi: 10.1590/0102-311XPT131623 (PMC11321612; doi:10.1590/0102-311XPT131623)
Supplement: Supplementary file 1 [file 1678-4464-csp-40-06-PT131623-s.pdf]

MATERIAL SUPLEMENTAR

Tabela S1 Número de casos incidentes de neoplasias malignas, todas as localizações, exceto pele não melanoma (C44) segundo registros de câncer de base populacional (RCBP), por ano, no período de 2007 a 2016.

**Região**	**Nome do RCBP (período de registro disponível no sistema)**	**2007**	**2008**	**2009**	**2010**	**2011**	**2012**	**2013**	**2014**	**2015**	**2016**
Norte	Acre (2010-2017)				696	716	836	607	746	735	645
	Belém (1996-2017)	2.627	2.723	2.667	2.798	2.955	3.003	2.976	2.921	3.234	3.046
	Manaus (1999-2013)	1.723	2.138	2.131	2.468	2.894	2.694	2.579			
	Palmas (2000-2013)	160	234	222	227	326	315	305			
	Rondônia (2015-2017)									2.501	1.630
	Roraima (2003-2010)	314	464	432	285						
Nordeste	Alagoas (2010-2011)				3.396	3.432					
	Aracaju (1996-2014)	1.127	1.090	1.130	1.223	1.311	1.383	1.458	1.317		
	Fortaleza (1990-2013)	4.689	5.080	4.955	4.123	4.694	4.964	4.767			
	João Pessoa (1999-2016)	1.309	1.295	1.332	1.473	1.374	1.527	1.541	1.730	1.861	1.923
	Natal (1999-2008)	1.759	1.759								
	Recife (1995-2016)	3.234	3.045	3.377	2.992	3.253	3.047	3.267	3.542	3.952	3.651
Sudeste	Angra dos Reis (2007-2016)	183	161	162	214	207	264	244	196	173	163
	Barretos (2000-2018)	877	950	927	941	989	1.064	1.033	1.060	1.146	1.060
	Belo Horizonte (2000-2017)	6.538	6.262	6.839	7.299	7.088	7.391	7.132	7.216	7.719	6.539
	Campinas (2010-2016)				3.551	3.545	3.466	3.493	3.276	3.542	3.660
	Espírito Santo (1997-2012)	1.501	1.521	2.624	2.981	2.851	2.807				
	Jahu (1996-2018)	339	357	358	316	352	354	353	357	347	345
	Poços de Caldas (2007-2014)	384	383	362	439	378	444	439	449		
	Santos (2008-2009)		1.516	1.637							
	São Paulo * (1997 -2015)	29.866	30.962	35.196	34.113	35.620	34.679	34.650	31.458	26.480	
Sul	Curitiba (1998-2016)	3.951	3.963	3.905	4.156	4.223	4.030	4.232	4.237	4.278	4.347
	Florianópolis (2008-2016)		1.412	1.647	1.670	1.742	1.654	1.980	1.812	1.940	1.809
	Porto Alegre (1993-2012)	4.724	2.928	3.284	4.708	4.795	4.011				
Centro-oeste	Campo Grande (2000-2003; 2008-2012)		1.773	1.910	1.903	2.069	1.614				
	Cuiabá (2000-2016)	1.229	1.105	1.078	1.413	1.405	1.536	1.648	1.696	1.743	1.540
	Distrito Federal (1999-2014)	3.792	4.714	4.472	4.454	4.456	4.188	4.650	5.363		
	Goiânia * (1988-2013)	3.320	3.371	3.337	3.063	3.314	3.031	2.857			
	Mato Grosso – interior (2000-2016)	1.865	1.622	1.731	2.218	2.602	2.576	2.823	3.042	2.851	3.130

Nota: nos anos sem informação para o RCBP as células foram mantidas em branco.

* Não utiliza o Sistema BasePopWeb (BPW).

Tabela S2 Indicadores de qualidade dos registros de câncer de base populacional (RCBP) para os registros dos casos de câncer exceto pele não melanoma no período 2007 a 2016.

Região	Nome do RCBP (período de registro disponível no sistema)	Meio diagnóstico			Localização primária desconhecida e mal especificada	Número de anos no período de estudo	Incluído na análise final
		%VM	%SDO	Sem informação (%)			
Norte	Acre (2010-2017)	64,63 *	34,47 *	0,06	6,44	7	Não
	Belém (1996-2017)	74,79	19,64	0,15	2,57	10	Sim
	Manaus (1999-2013)	67,42 *	29,10 *	1,71	3,21	7	Não
	Palmas (2000-2013)	80,55	17,72	0,28	1,17	7	Sim
	Rondônia (2015-2017)	55,80 *	36,38 *	0,90	3,73	2	Não
	Roraima (2003-2010)	82,68	9,70	5,48	4,62	4	Sim
Nordeste	Alagoas (2010-2011)	77,04	17,38	0,00	3,94	2 *	Não
	Aracaju (1996-2014)	92,36	3,96	0,01	1,26	8	Sim
	Fortaleza (1990-2013)	80,59	13,19	4,89	3,30	7	Sim
	João Pessoa (1999-2016)	82,12	12,02	3,76	5,23	10	Sim
	Natal (1999-2008)	85,70	13,67	0,40	4,09	2 *	Não
	Recife (1995-2016)	72,18	12,65	0,70	2,97	10	Sim
Sudeste	Angra dos Reis (2007-2016)	61,92 *	29,44 *	3,76	4,02	10	Não
	Barretos (2000-2018)	82,93	15,28	0,54	3,13	10	Sim
	Belo Horizonte (2000-2017)	92,11	7,37	0,35	4,93	10	Sim
	Campinas (2010-2016)	84,95	11,82	0,66	1,95	7	Sim
	Espírito Santo (1997-2012)	69,25 *	29,99 *	0,11	2,61	6	Não
	Jahu (1996-2018)	93,96	0,63	0,14	4,95	10	Sim
	Poços de Caldas (2007-2014)	90,79	7,66	0,09	2,01	8	Sim
	Santos (2008-2009)	79,26	17,48	1,90	2,12	2 *	Não
	São Paulo ** (1997 -2015)	85,58	1,15	0,00	6,65	9	Sim
Sul	Curitiba (1998-2016)	81,41	11,74	0,05	2,99	10	Sim
	Florianópolis (2008-2016)	90,62	5,13	1,13	2,03	9	Sim
	Porto Alegre (1993-2012)	60,35 *	30,84 *	0,71	3,49	6	Não
Centro-oeste	Campo Grande (2000-2003; 2008-2012)	82,85	10,84	0,04	3,30	5	Sim
	Cuiabá (2000-2016)	84,13	13,42	0,26	2,38	10	Sim
	Distrito Federal (1999-2014)	76,68	19,94	1,58	3,01	8	Sim
	Goiânia *** (1988-2013)	90,47	8,19	0,41	2,68	7	Sim
	Mato Grosso – interior (2000-2016)	64,33 *	33,63 *	0,60	5,61	10	Não

* Valor fora do parâmetro de qualidade definido;

** Não utiliza o sistema BasePopWeb (BPW);

*** Não utiliza o sistema BPW – informações representam dados disponíveis (a base divulgada não apresenta informação para a variável meio diagnóstico, nos anos de 2008, 2009 e 2012.

%SDO: percentual de casos identificados somente pela declaração de óbito; %VM: percentual de casos com diagnóstico por verificação morfológica, citologia, histologia do tumor primário ou da metástase.
